# The botanical multiverse of Peter Barlow

**DOI:** 10.1080/19420889.2019.1575788

**Published:** 2019-02-03

**Authors:** Nigel Chaffey, Dieter Volkmann, František Baluška

**Affiliations:** a College of Liberal Arts, Bath Spa University, Bath, UK; b IZMB, University of Bonn, Bonn, Germany

**Keywords:** Development, morphogenesis, patterns, patterning, roots, stomata, modelling, cell biology, cytoskeleton, cell walls, ethylene, ecology, gravity, lunisolar, plant neurobiology, Cell Theory, Cell Body, fossil plants

## Abstract

Dr Peter Barlow, who died in 2017, was one of the most respected botanists and biologists of the latter half of the 20th Century. His interests covered a wide range of plant biological topics, e.g. root growth and development, plant cytoskeleton, effects of gravity, plant intelligence, pattern formation, and evolution of eukaryotic cells. Here we consider Peter’s numerous contributions to the: elucidation of plant patterns; understanding of root biology; role of the plant cytoskeleton in growth and development; influence of the Moon on terrestrial vegetation; Cell Body concept; and plant neurobiology. In so doing we attempt not only to provide an overview of Peter’s important work in many areas of plant biology, but also to place that work in the context of recent advances in plant and biological sciences.

## Introduction

“I come to bury Caesar, not to praise him“

[Words spoken by Marc Antony in *Julius Caesar*, Act 3 Scene 2 by William Shakespeare]

(King and King, 2004–2016)

**Figure 1.**
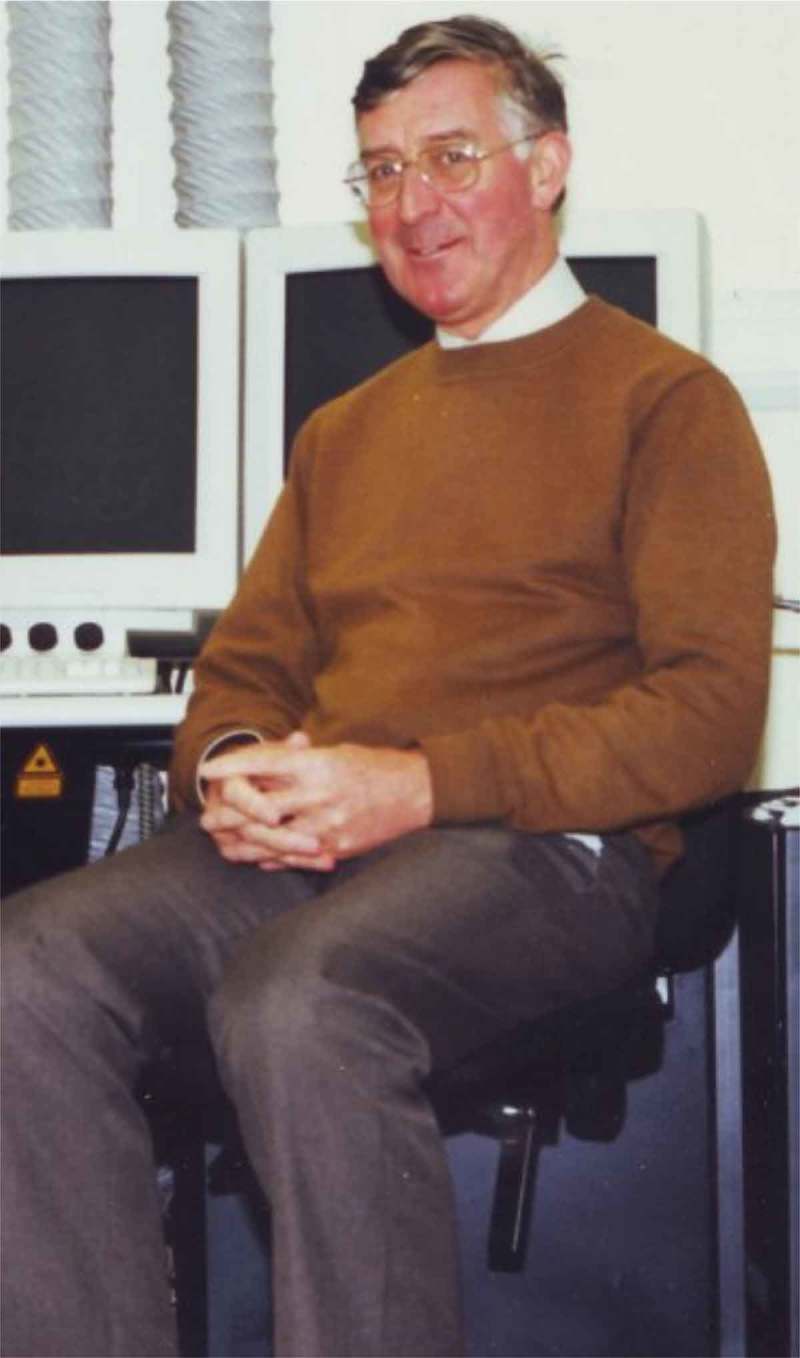
Peter beside the Long Ashton Research Station confocal microscope that contributed so much to his investigations into the cell biology of wood formation in trees.

In January 2017 the world of plant science lost one of its most insightful practitioners, Dr Peter Barlow (Figure 1). To date no formal appreciation of his researches – nor therefore of his rightful place in the pantheon of plant biology practitioners – has appeared since his passing. To our knowledge the only official recognition of the great man’s demise is a Special Issue of the *Annals of Botany* [*SIAB*] on Developmental Plant Cell Biology (Annals of Botany Office, 2018). Although that SIAB contains Reviews, Viewpoints, Research in Contexts, and Original Articles on topics that were close to Peter Barlows’s topics of interest in plant biology, that collection of modern-day researches makes no attempt to place Peter’s contributions into its rightful context.

Peter was fond of using quotes – providing they were apt and appropriate – to enhance his own writings. Choosing to begin this article in that way is therefore carrying on that Barlow ‘tradition‘. But, unlike Marc Antony who is alleged to have uttered the words quoted above, we *are* here to ‘praise‘ Peter and honour his memory by considering his numerous contributions to plant science. However, the Barlow publications cited in this appreciation constitute neither a detailed nor exhaustive analysis of all of Peter’s published work. Rather, we attempt merely to place his important contributions to botany in the context of the current research by other practitioners as contained in the *SIAB*. This article is also not a full obituary of the man and his achievements, but a personal reflection of him by three people who have been long-time collaborators and friends of Peter.

*A short botanical biography of Peter Barlow* [14th August 1942 – 26th January 2017]

Peter read Botany at the University of St Andrews (Scotland) and gained his Batchelor of Science degree there in 1965. Thereafter he went on to post-graduate study at Balliol College (University of Oxford, UK) and was awarded his Doctor of Philosophy in 1969 for his thesis entitled “Organisation in root meristems“.

Somewhat surprisingly for such a well-known figure in the botanical world, Peter then held post-doctoral positions in biomedical-oriented research (but an area of research to which he returned in later years – see **Concluding remarks**). First at the Paediatric Research Unit of Guy’s Hospital Medical School (London, UK) – working with Prof. PE Polani FRS on the effect of human X-chromosome aneuploidy on cell division, and then the Department of Zoology (University of Oxford, UK) – with Dr CF Graham FRS, studying the embryogenesis and trophoblast formation of the mouse. But, he didn’t stay away from botany for long, and in 1971, after a term lecturing on plant development at Unversidad de La Plata (Argentina), Peter was invited to become a founder member of the Unit of Developmental Botany (under Director Prof. PW Brian FRS) at the University of Cambridge (UK). He remained there for 7 years before another 7-year stint at the Letcombe Laboratory (Wantage, near Oxford), and then worked at Long Ashton Research Station (near Bristol, UK). On his retirement from Long Ashton, Peter became an Honorary Research Fellow in the School of Biological Sciences at the University of Bristol (Bristol, UK), a post he held until his passing in 2017.

Amongst his awards and honours was the conferment of his Doctor of Science degree from St Andrews University in 1992, and, in 2001, he was the proud recipient of both the ‘For Merit’ medal of the Slovak Academy of Sciences, and the Jozef Ludevít Holuby medal of the Botanical Society of Slovakia. Peter served on editorial boards of many academic journals during his career, and most recently was associated with *Plant Biosystems, Plant Root*, and *Plant Production Science*.

So much for a brief Barlow biography. What about the scientific work he undertook, and which can be considered to be both his contribution to plant science, and his legacy? Arguably, the best way to do that, and one which also demonstrates the relevance of his various insights into, and contributions towards, elucidation of several botanical phenomena can be gained from the diverse contributions in the Special Issue of the *Annals of Botany* (*SIAB*) organized to celebrate Peter‘s own work (Annals of Botany Office, 2018).

As a reminder of Peter’s wide range of interests, the invitation for contributions to the *SIAB* had a focus upon: “root growth and development, the quiescent centre, plant cytoskeleton, polarity, gravitational plant biology, plant signalling and intelligence, biological rhythms, pattern formation and modelling, and evolution of eukaryotic cells“ (Annals of Botany Office, 2017).

And as an apt, scene-setting quote for what follows, we offer these words about Peter.

“Peter would be at his microscope, counting his cells, realising patterns and thinking deeply about where his work was leading. In an “ivory tower” some might have said, but it was not so. If tower there was it was one into which he climbed to launch himself onto, what seemed at first to be, flights of fancy. But My! His vision on his flights picked up clues to the universal significance of the stem cells of the quiescent centre, of the necessity for cell death and its importance, of plant ‘neurology’ and ‘immunology’, of the influence of lunar cycles on the behaviour and properties of plants. Much of what he inferred from these clues, as arcane as they were to the rest of us, has proved to be more than half right. To be more than half right is probably as much as any of us can aspire to.“

[Prof. David T Clarkson, friend and colleague of Peter Barlow]

## Peter’s plant patterns

Peter had the knack – albeit one honed by many years of careful and patient observation, detailed study and reflection on the meaning of sequences, etc. – of recognising patterns in biological phenomena. This is particularly evident in his more recent work on cell divisions and tissue patterning amongst the multiple cell types of the secondary vascular tisues of trees [1,2]. But his interests in patterns ranged far and wide, from heterocysts of *Anabaena catenula* (a cyanobacterium) [3], to meristems in *Psilotum nudum* (a so-called ‘whisk fern) [4], rhizophore apices of *Selaginella* (a lycopsid), the meristem and cap of the root of *Zea mays* (a grass) [5], cortical diaphragms in *Thalassia testudinum* (a seagrass) [3], and stomatal patterning in species such as *Arabidopsis thaliana* (a crucifer), *Begonia peltatifolia* (a begonia), and *Cinnamomum camphora* (camphor tree) [6].

Those studies often used sophisticated methods of analysis such as ‘bootstrap‘ L-systems, as exemplified in his long-standing collaboration with Jacqueline and Hermann Lück [3,4,6–11]. This marriage of mathematics and modelling to the ‘old-fashioned‘, traditional observational botany approach [12] was a particular forte of Peter‘s that is also particularly evident in his lunisolar researches [see **Extra-terrestrial influences on plant-life**]. In many respects, this patient, careful approach brings to mind the quote attributed to Louis Pasteur that “*in the fields of observation chance favors only the mind which is prepared*“ [13,14]. How many of us may have looked at biological phenomena, but not really *observed* and, still less, understood what we are seeing? Or, as eloquently expressed by Daniel Mazia (originator of the Cell Body concept [15,16]), “*The gift of the great microscopist is the ability to think with the eyes and see with the brain*“ [17].

It is entirely fitting therefore that the *SIAB* has important contributions to elucidating patterns in plants, from workers examining geologically-distant evolutionary dimensions in fossil moss leaves [18], to those investigating extant taxa in a study of the comparative development of floral spurs in toadflax [19], and stomatal development in begonias [20]. Grasses are also well-catered for – *Zea mays* was a favoured experimental organism of Peter’s, which he used many times during his career (for such work as ultrastructural study of its quiescent centre [21], endoreduplication in metaxylem cells [22], root morphogenesis [23], cytoskeleton studies [24], gravitropism [25], and investigations of the impedance of soils to root growth [26]). Appropriately, in the *SIAB*, we have developmental study of fusoid cells in grass leaves [27], and the review of cross-talk between cells during formation of the subsidiary cells in maize [28].

Leandro et al. [27] investigate fusoid cells, important features of the blade of certain grass species, but which, despite having acknowledged systematic value, are little understood structures in terms of their taxonomic distribution amongst the Poaceae and their functional role within the grass leaf. Using detailed microscopic investigations the authors chart the development of these features in 20 species in three families – Poaceae, Flagellariaceae, and Joinvilleaceae (the latter two representing the earlier diverging and derived branches within the Graminid clade and Poaceae). In all examined taxa of the Poaceae, fusoid cells originate from the ground meristem, as do the colourless cells in *Joinvillea ascendens* (Joinvilleaceae), and both these types of mesophyll cells have a strongly similar ontogeny suggesting that they are homologous. Leandro et al. also present results suggesting that, at least in young grass leaves, fusoid cells play a role related to synthesis and storage of starch granules at early stages of development [27].

At first sight, this study [27] is of a topic which may seem arcane, and of limited relevance to real-world concerns. But, this ‘left-of-field‘ sort of study brings to mind Peter’s own seemingly off-kilter work on the developmental anatomy of Ginkgo [29], which develops curious downgrowths – known as chi-chi – from aerial parts of the stem. As with Leandro et al. fusoid cell study [27] and the relevance of those structures to photorespiration [30] or partitioning of photosynthate within cells of grass leaves and cereal crops more specifically, Barlow & Kurczyńska’s study [29] provides an anatomical and developmental study which gave insights into a mode of elongation growth that is an *alternative* to that which is usually considered to be the preserve of apical meristems.

Ivanov et al. have taken up the considerable challenge of trying to decipher cellular patterning in leaves of fossil mosses [18]. In particular they examined whether the structure and variation of the areola of those ancient taxa are comparable to those of modern-day *Sphagna* taxa. Images of the fossil leaves were taken and analysed via computer and showed that the fossil areolation pattern is identical in its basic structure to both that of modern *Sphagnum* spp. *and* Palaeozoic protosphagnalean mosses. However, the cell divisions that give rise to the same features in extant and extinct mosses are different. This insight leads the authors to conclude that the fossil protosphagnalean mosses had the ability to switch their development of leaf areolation between a pathway unique to *Sphagnum* and another that is common to all other mosses. Not surprisingly, this new understanding of the ontogeny of these structures has caused the authors to re-evaluate the systematic significance of such diagnostic characters in these Palaeozoic plants. This paper is particularly important because it shows what can be done with appropriate techniques – which were specially developed to address the problem being investigated – to elucidate patterns in taxa that are approx. 265 millions of years old and contribute to a better understanding of the evolutionary history of land plants. In this respect, the remarkable value of anatomically preserved fossil bryophytes is to be admired and appreciated [31]. But, again unless one really observes what is there, many patterns might otherwise go unnoticed. Ivanov et al. paper [18] is therefore a great example of that typical Barlow characteristic of both *observing* and having a *mind that is prepared* to understand what is seen.

Peter’s interests in plant anatomy with a focus on cell patterns, cell division planes, and interplay of cell division and cell elongation are represented by three papers in the *SIAB*. Nectar spurs are tubular outgrowths of floral organs which either contain, or give the appearance of containing, nectar. With their role in providing energy-rich materials for pollinating organisms, nectar spurs are seen as a ‘key innovation‘ that can lead to rapid speciation. However, despite their ecological importance, the developmental anatomy of these structures has been little studied. Within the genus *Linaria*, Cullen et al. [19] focus on *L. becerrae* and *L. clementei* and report that cell *number* is 3 times higher in the former long-spurred species, but cell *length* is only 1.3-times greater. Overall, anisotropy – directed cell *expansion* – of mature cells is similar for both species. They therefore infer that evolution in nectar spur length in *Linaria* is largely explained by differences in cell number and hence cell division. This conclusion contrasts with studies in *Aquilegia* where cell *anisotropy* gives rise to variation in this structure. Thus, different species may have evolved the same structures but using different mechanisms.

Two contributions in *SIAB* examine stomatal development. Rudall et al. [20] provide a detailed light and transmision electron microscope study of the cellular events involved in the development of stomata in *Begonia* leaves. Stomata in this taxon are helicocytic [with a helix, of four or more subsidiary cells surrounding the guard cell pair, whose development is characterised by an inward spiral of cells surrounding a central stomatal pore. Although relatively rare, this feature occurs in some drought-tolerant angiosperm species. Furthermore, in some thick-leaved *Begonia* spp., stomata are not only helicocytic but also clustered into groups spaced apart by at least one cell. That non-contiguous, helicocytic stomatal patterning is carefully scrutinised by Rudall et al. [20]. in their elegant, traditional ontogenetic study.

At the other end of the spectrum of analysis of plant cell biological phenomena, Apostokalos et al. [28] review structural and molecular mechanisms relating to cell polarization that lead to asymmetric cell divisions underlying stomata formation in maize leaves. This cell polarization is based on a dynamic cytoskeleton, including F-actin bundles/networks, as well as microtubules polarizing subsidiary cell mother cell (SMC) cytoarchitecture towards the guard cell mother cell (GMC). Asymmetric SMC cell division is preceded by polar migration and anchoring of the SMC nucleus associated with a unique cage of microtubules (resembling the Cell Body complex – see **Challenging the *Status Quo***). Apostokalos et al. [28] discuss extensively signaling complexes and molecules regulating this SMC polarization induced by the adjacent GMC, including Rho-like GTPases and leucine-rich repeat receptor-like kinases (LRR-RLKs), auxin and its transporters, reactive oxygen species (ROS), and signaling phospholipids controlled by phosphatidylinositol- 3-kinase and phospholipases (PLC and PLD). Control of the actin cytoskeleton dynamics via the SCAR–WAVE regulatory complex, and dynamism of microtubules via katanin and MAPKs are also discussed. That study leads to a consideration of another of Peter’s favourite topics of research, the plant cytoskeleton.

## Peter and the cytoskeleton

Peter exploited indirect immunofluorescent antibody approaches for localisation of tubulin, actin, and myosin components of the plant cytoskeleton and studied aspects of their biology in grass and tree species. These studies primarily occupied Peter’s attention in a highly productive 10-year period from approx. 1992 to 2002, which can be broadly divided in to two distinct, but overlapping, phases.

### Peter’s maize root cytoskeletal work

Peter used maize as his experimental organism of choice for many of his plant studies. It seems likely that his love affair with this important cereal began when he joined the laboratory of Frederick Clowes at the University of Oxford, to undertake his doctoral studies. This is evident in seven (of the eight) papers he published from his DPhil thesis [32–38]. That catalogue of publications was also an indication of the prodigious output that was to be a hallmark of Peter’s work rate throughout his career.

Peter continued to use maize as his preferred object of study throughout the 1970s and 1980s, and his laboratory was the first in the world to use sections of maize roots embedded in Steedman’s wax for indirect immunofluorescence of tubulin to visualize microtubules in cells within intact tissues of a plant organ. It is important here to acknowledge the crucial role played by Jill Parker (née Adam) in developing the Steedman‘s Wax technique for those maize root studies [39,40]. Jill provided long-standing technical support to Peter at Long Ashton Research Station, and her contribution to this work is more formally celebrated by her co-authorship of several seminal maize cytoskeletal papers [39–43]. This innovation led to publications that documented specific patterns of cortical and endoplasmic microtubules associated with cell growth and tissue differentiation in roots of maize [39], tissue-specific responses of the microtubular cytoskeleton in cells of cold-treated roots of maize [42], a role of gibberellic acid in orienting microtubules and regulating cell growth polarity in the maize root cortex [43], and the involvement of microtubules, ethylene and gibberellin in the differentiation of cellular behaviour in post-mitotic growth zones in maize roots [41]. Microtubules were Peter’s main cytoskeletal component of interest [25,39,41–43], but he also investigated actin and myosin [44].

### Peter’s tree cytoskeletal work

Although Peter never lost interest in the cytoskeleton within primary plant tissues, his cytoskeletal studies also extended to the cell biology of secondary vascular tissues. In 1994, Peter and John Barnett [University of Reading, UK] were co-recipients of a BBSRC grant to investigate the role of the cytoskeleton in the vascular cambium and wood formation in trees. The work proposed was challenging, so much so that one reviewer of the grant proposal expressed doubts that the cytoskeleton could be observed in such a difficult tissue. Well, Peter confounded the critics; the cytoskeleton could be observed in these tissues. This led to a very successful collaboration that extended over 7 years between Peter, John and their post-doctoral worker, Nigel Chaffey. But, in typical Barlow fashion, Peter didn’t study the obvious, readily-accessible, above-ground stem or branches of trees, but ploughed his own furrow in investigating the cytoskeleton in *roots* of *Aesculus hippocastanum*, an arborescent angiosperm.

The success of that work resulted in both Peter and Chaffey becoming major contributors to the Woodform project led by John Barnett. This EU-funded FAIR shared-cost research project entitled “Wood formation processes: the key to improvement of the raw material”, was an international co-operation between research groups in the UK, Sweden, France, and Germany and examined both wild-type and transgenic hybrid aspen (*Populus tremula* x *P. tremuloides*), principally the stems this time.

That extended collaboration with Chaffey, and usually also John Barnett, and Peter‘s pioneering studies of the cytoskeleton of the vascular cambium and its derivatives led to an impressive catalogue of ‘firsts’: documentation of involvement of microtubules [45], actin microfilaments [46], and myosin [47] in differentiation of secondary vascular tissues; the proposed adoption of hybrid aspen as a model system for wood cell biological investigation [48]; development of new techniques for visualisation of the cytoskeleton in secondary vascular tissues [49]; identification of a putative plant muscle involved in development of the bordered pits of vessels and tracheids in angiosperm and gymnosperm trees, respectively [47]; recognition of the potential for co-operation between microtubules, actin and myosin in cell division within cambia [47]; the proposal of a cytoskeleton-mediated, supra-cellular, 3-dimensional transport and communication pathway within and between the living cells of the secondary vascular system [50]; identification of a seasonal cycle of microtubule orientation within the cambium during active and resting periods [51]; and the suggestion that the arrangement of microtubules, but not actin microfilaments, indicates determination of cambial derivatives to a particular developmental pathway [52].

In the *SIAB*, Peter’s cytoskeletal interests are represented by two Original Articles, both of which focus upon actin. Sahi et al. [53] consider the ARP2/3 complex that controls F-actin rearrangements that are relevant to plant morphogenesis in connection with cell walls and polar auxin transport. Their work demonstrates inhibition of polar auxin transport and disruption of auxin distribution in cells of ARP2/3 mutant lines. This study strongly implies a morphogenetic roles of the ARP2/3 complex in cell wall synthesis and polar auxin transport. Vaškebová et al. (2018) analyzed the roles of *ACTIN2*, which is essential for bulge site selection and tip growth of root hairs [54]. Importantly, they investigated aspects of the cytoskeleton and plant growth in addition to effects of this root-hair deformation in the *der1–3* mutant of Arabidopsis. They found that actin *filaments* in roots, hypocotyl and cotyledon epidermal cells were shorter, thinner and more randomly-arranged than in wild tpe plants, and actin *bundles* were shorter with altered orientations. Although cortical microtubule organisation in root cells was not affected, the wavy pattern of root growth in the mutants was associated with higher frequencies of shifted cell division planes, which is consistent with shifts in positioning of microtubule-based pre-prophase bands and phragmoplasts. Vaškebová et al. [54] therefore propose that the *ACT2* mutation in the *der1–3* mutant doesn’t only affect root hair formation, but also has effects more generally on plant growth and development *via* the actin cytoskeleton. Such work continues to adds to our growing appreciation of the multitude of developmental processes in which the cytoskeleton – in all its myriad components – is involved in the life of the plant. And that elucidation continues as the role of the cytoskeleton is elegantly demonstrated in its contribution to the symbiosome that results from infection of roots by *Rhizobium* bacteria in the legume nitrogen-fixing partnership [55]. And which underground connection leads neatly to the next section.

## Subterranean interests – roots and more roots…

One of Peter’s long-time research interests was roots. Indeed, roots – and, somewhat presciently, vascular tissue differentiation (a subject explored above in **Peter and the cytoskeleton**) – were the topic of Peter’s first published paper [56]. In this article we have already highlighted aspects of that work in **Peter and the cytoskeleton**, and **Peter’s plant patterns** sections, and it will reappear in **Extra-terrestrial influences on plant-life**, and **Plant ‘neurobiology‘, pushing the boundaries**. Indeed, so important were roots to Peter that it seems entirely appropriate that one of the last papers he was working on was an obituary of FAL Clowes [57], Peter’s DPhil supervisor and noted root researcher who coined the term root apex “quiescent centre“ [58].

### Cell reproduction in roots

Root extension is dependent upon cell production at the apical meristem and the subsequent growth and differention of those daughter cells. And meristematic activity and cell division of roots was an early pre-occupation of Peter as seen in papers such as: Barlow [59], where he mused on the stem cell-like properties of such cells; Barlow [1] in which he examined endoreduplication in metaxylem cells of corn, the effects of temperature on the cell cycle [60]; development of a technique for excising and squashing roots the better to analyse the cell cycle in the quiescent centre [61]; and many papers on aspects of the biology of the quiescent centre [62–64]. He also studied nucleotypic effects on cell cycle parameters [65].

This aspect of Peter’s work is represented in the *SIAB* by Zhukovskaya et al. [66] Information on cell cycle duration (*T*) in the root apical meristem illuminates growth, development, and evolution of that plant organ. Obtaining estimates of that parameter are therefore important. One of that paper’s authors had previously proposed a simple method for evaluating *T* which was based upon the dynamics of root growth, and termed the RCP (Rate-of-Cell-Production) method. The other main technique for determining *T* is the ^3^H-thymidine method. However, and although classical, that radioactive method is both laborious and time-consuming. In order to speed-up ascertainment of *T*, Zhukovskaya et al. [66] present a global analysis of 73 angiosperm species using the RCP method, and compare the derived *T* values with those from the ^3^H-thymidine method. They find that, in most species examined, *T* values obtained by both methods were nearly identical, and conclude that the RCP method enables cell cycle duration in the root apical meristem to be both rapidly and accurately determined. Accordingly, the authors recommend the RCP approach for its simplicity – relative to the tritiated thymidine method – and which can be performed using live or fixed roots, and propose its use in the fields of phenomics, evolutionary ecology, and other plant biology studies.

### Lateral roots

Roots don’t just increase in length due to the activity of the apical meristem, they may also branch and send out lateral roots. Peter investigated these aspects of root growth too in cultured root axes of tomato [67], and explored the possibility that their spacing may be multiples of a ‘fundamental distance’ [68]. He did not neglect the external environment in which roots grew when investigating the effect of nitrate concentrations on root branching in Arabidopsis [69], which has subsequently been developed by others into a broader consideration of root system architecture and the effects of edaphic and endogenous signals [70]. The *SIAB* has two contributions that concentrate on aspects of the biology of lateral roots.

Ilina et al. [71] analyzed lateral root initiation and formation within the parental root meristem in *Cucurbita pepo*. The auxin signaling-responsive promoter DR5 was used to visualise cellular auxin response maxima. Importantly, auxin response maxima were observed *before* the first formative (asymmetric) cell divisions were seen in the pericycle and endodermis. Chiatante et al. [72] reviewed the roles of pericycle and vascular cambium in the development of lateral roots. Besides auxin signaling and transport, they also focused on peptide signaling pathways and the roles of critical transcription factors, not only in annual plants but also in herbaceous and woody plants.

### Roots and the external environment

In the wider environment beyond the hosting plant, roots experience many abiotic factors that impact upon their biology. We’ll encounter more of Peter’s interests below in such matters in the **Extra-terrestrial influences on plant-life**, but he also investigated some more down-to-earth issues. For example, he investigated the effects of sea water on roots of *Prosopis alba* [73], in collaboration with colleagues from South America where this species is a valuable resource in arid areas, providing food, medicines and fuel, and can be used to control erosion [74]. That experimental work has relevance in understanding how *Prosopis* might cope with salinity stress in the wild, which is an environmental stress experienced by plants in many parts of the world [75]. Another major issue that is global is how plants – especially their roots – respond to elevated concentrations of heavy metals in the soil [76,77]. In the *SIAB*, this important interaction is examined in two research articles.

Working with copper (Cu), a major heavy metal contaminant of soil [78], Kováč et al. [79] showed that radish roots exposed to elevated Cu concentrations develop a subero-lignified apical deposit (SLAD). The SLAD is a unique structure consisting of modifications to the cell walls of the root’s central cylinder that are encircled by a short cylinder of prematurely-suberized endodermal cells. The SLAD starts to form, after root elongation has ceased, in both primary and lateral roots, and is associated with xylem differentiation and root-branching close to the root apex. Although the SLAD appears to be a root-specific response to elevated Cu concentrations, future studies are needed to investigate its exact role in any root adaptation to heavy metal stress.

Kohanová et al. [80] considered cadmium (Cd), another soil-contaminating heavy metal [81], and the responses of both roots and aerial parts of the experimental plant. They examined responses of root-hair deficient, and hairy root mutants of Arabidopsis and compared them with wild-type Columbia plants. Cd inhibited plant growth, and reduced root length, the number of lateral roots, and root hairs in Arabidopsis. Importantly, shoot Cd accumulation was shown to be positively correlated with root hair abundance, but only at 10 µM Cd. Treatment with 100 µM Cd resulted in development of suberin lamellae closer to the root tip, which was associated with *restricted* Cd accumulation in the shoots. The work of Kohanová et al. [80] underlines the importance of root hairs, which has also been highlighted in respect of both the responses of roots to challenge by pathogenic strains of *Pseudomonas syringae* [82], and their role in carbon input *into* the soil [83]. Consequently, anything in the soil environment – such as heavy metals – that affects root hair development may have profound consequences, not only for the health of the affected individual plant, but also its contribution to the greater ecology of the area, and globally in terms of carbon cycling.

Roots grow through the soil away from the host plant and are likely to interact with other roots, in the substrate. A typically idiosyncratic take on this was Peter’s work looking at plant roots and fungal hyphae [84]. However, rather than examine the more obvious fungus/higher plant connection of mycorrhiza – an ancient mutualism [85,86] of importance both to land plant’s evolutionary success and to continued and continuing productivity of the host plant [87] – they compared the ‘oriented collective motion‘of plant roots to the ‘swarming‘of fungal hyphae. Barlow and Fisahn concluded [84] that so-called ‘active swarming‘ of these evolutionarily distant life-forms might indicate that swarming is a fundamental property of organisms arrived at by an evolutionary convergence, and that it may also indicate a capacity for cooperation between mobile organismic elements that benefits the species and its gene pool.

Extending a consideration of roots to the natural setting of a forest of different tree species, but with an abiotic edaphic dimension, is the *SIAB*’s paper by Wang et al. (2018) [88]. Interested in the interactions between heterogeneous forests and their biophysical environment, these authors studied the impact of local climate upon tree physiology. To do so they examined tree roots, and measured the number that were growing, their elongation and mortality through temporal and spatial variations in soil temperature and water potential [88]. They made these measurements every month over 4 years, using rhizotrons. Amongst their findings were that mean daily root elongation rate was not correlated with soil water potential but was significantly and positively correlated with soil temperature, and peaked in spring. Root longevity was dependent on altitude and the season in which roots were initiated, and root diameter was a significant factor explaining much of the variability observed. The finest roots usually grew faster and had a higher risk of mortality in gaps than in closed forest, and at 2000 m, they had a higher risk of mortality compared to the lower altitudes [88]. Overall, heterogeneous forest structure and location play a significant role in determining root demography in temperate, montane forests, mostly through impacts on soil temperature.

### Roots interacting with the soil

Roots don’t just interact with factors *in* the soil, they are also affected *by* the physical nature of the soil itself, and gravity. Peter had great interests in both these issues. For example, he not only modelled root growth and bending in two dimensions [89], but also examined how intact [26] and decapped [90] roots fared when growing in compacted sand. Peter also had an abiding interest in the root’s detection of, and response to, gravity [21,91,92]. Both of those facets of Peter‘s study of roots are represented in the *SIAB*.

Potocka and Szymanowska-Pułka’s review [93] the morphological responses of plant roots to mechanical stresses. Several modifications in root morphology are known: decreased root lengths; radial swelling of root apices; and enhanced sloughing of root cap cells. All these responses are directly, or indirectly, connected to ethylene emission and signaling. Continuing with an emphasis on this plant growth regulator, Dreyer and Edelmann examined [94] ethylene emission in intact maize roots as compared with those devoid of their root caps. They report that intact root caps are needed to overcome mechanical resistance of soil, which appears to be mediated via specifically-induced emission of ethylene [94]. No emission of ethylene was observed in decapped roots under the same testing conditions. Both of these papers further underline the important role of this hormone in plant growth and development [95].

## Extra-terrestrial influences on plant-life

Although much of Peter’s work examined the internal milieu of plants, he was also interested in the effects of external factors. This is particularly well illustrated in his work aimed at predicting the environmental thresholds for cambial and secondary vascular tissue development in tree stems [96]. But, allthough there is much to interest the inquisitive botanist on Earth, Peter was not confined to earth-bound matters, he also had at least one eye on an extra-terrestrial dimension. Interest in such matters was underlined by his role as Vice-Chairman of COSPAR (Committee on Space Research) Section F 1.1 Life Sciences (1997–2001), and his Membership of the Life and Physical Sciences and Applications Advisory Committee, British National Space Centre (1998–2001). His research interests in this sphere stemmed from his work on root gravitropism, in such publications as Barlow [97] and Barlow et al. [98].

More recently, Peter’s interests turned towards the moon and the influence that this satellite may have on biological phenomena on Earth [99]. That conference presentation was followed up by many papers investigating the influence of the moon on such plant phenomena as leaf movements [99–101], stem elongation [102], fluctuations in diameters of tree stems [103], root growth [104–106], emissions of biophotons by seedlings [107–110], and chlorophyll fluorescence [111]. Not constrained by gravity, Peter was also developing ideas about extra-terrestrial influences and plant bioelectricity [112].

Large-scale tides caused by the moon are familiar to us on Earth as the regular fall and rise of the oceans at the coast, and affect many aspects of animal biology [113]. The micro-scale events that Peter was investigating are a reminder that it’s not only the big, clearly-visible external factors that affect life, but it is also the small-scale phenomena, which are often barely detectable by the unaided eye – or to the untrained or unprepared observer – that may have impacts upon plant biology as well. Had he been alive at the time, no doubt Peter would have regarded the award of the 2017 Nobel Prize in Physiology or Medicine to Jeffrey Hall, Michael Rosbash and Michael Young for their discoveries of molecular mechanisms controlling the circadian rhythm [114,115] with some wry amusement. For, in his Foreword to Gunter Klein’s *Farewell to the Internal Clock* [116], Peter was already musing on whether we shouldn’t be bidding the *internal* clock adieu, and welcoming in an era where it is replaced by the *lunar* clock [117].

Appropriate appreciations of Peter’s contributions to such ‘extra-terrestrial influences‘ on Earth-bound vegetation are contained within several contributions in the *SIAB* that focus on the roles of lunisolar tidal acceleration on several aspects of plant behaviour. Together those articles develop the notion that certain aspects of plant growth and physiology are mediated by lunisolar gravity fields.

Fisahn’s Viewpoint provides an important update on the question of whether there are ‘tides within trees‘, and considers changes in tree stem diameter and electrical potentials. In particular it shows that a synchronism exists between the timing of turning ponts in the lunisolar tide and the direction of extension changes within the tree. Whilst caution is still needed in terms of ascribing causality, these findings do suggest the posibility that dilations in stem diameter and electrical stem potentials may be somehow influenced by the lunisolar gravitational acceleration.

In another Viewpoint article, Fisahn et al. [118] show that circadian rhythms of Arabidopsis root elongation rates closely follow variations of lunisolar gravity fields. They also extend that work into the realm of quantum gravitational effects by proposing the intriguing notion that this influence may act via modulating the ‘coherent state‘ of water molecules within individual cells and their cell walls [118]. While that hypotheseis awaits more experimental investigation, an important implication from this study is that care should be taken in choosing – and accurately recording – when laboratory experiments are undertaken with young Arabidopsis seedlings.

Another paper that reports new data and findings on control of plant growth and movements via the Moon that are mediated by lunisolar gravity fields, which wasn’t part of the *SIAB* but which has been published in honour of Peter Barlow, is that by Zajączkowska et al. [119]. That article presents a detailed analysis of the nutations (non-autonomous movements) of stem tips of peppermint seedlings and concludes that they are significantly affected by the gravitational force of the moon. In that instance, Zajączkowska et al. [119] interpret such movements in terms of plant optimisation processes in response to imposed external environmental gradients. Interestingly, this phenomenon is only apparent in young stems; older stems don’t appear to exhibit this lunar-dependency. This finding may represent a legacy of a time long distant in the planet’s geological past when the Earth and moon were much closer together [120,121], and such interactions may have been stronger and more influential in plant growth and development. The work by Zajączkowska et al. [119] also argues for greater attention to detail in stating clearly in publications at what stage of the plant’s life cycle lunisolar studies are performed – such ancient legacy-behaviours might not be present or detectable in later stages of growth.

Gallep et al. [122] present an important review of Peter’s achievements with respect to the effects of lunisolar tidal forces on plants, including spontaneous ultra-weak emissions from plants. Importantly, they show how his extra-terrestrial interests developed naturally from his more Earth-bound pursuits, which give insights into Peter’s thinking on these matters. That paper also presents new data relating to UPE [ultra-weak photon emission] from coffee seedlings, over the whole germination period of 2 months, which spans the passage of two lunar cycles. The data support the notion that gravimetric tide minima and maxima affect the UPE of seedlings, but, as seedlings mature, the periodic components of the gravimetric tide become gradually less pronounced. A particular strength of this paper is that Gallep et al. [122] consider an explanation of how small changes in local gravity can have an effect on the growth of seedlings. And they propose a mechanism for sensing microgravity forces involving amyloplasts (which is consistent with Earth-bound graviperception by roots), or other dense structures [122].

Their suggestion involves mechanoreceptive molecular mechanisms that are not localized within one cell only, but have a ‘supracellular’ dimension [122]. Arguably, already anticipated by Barlow [123], Gallep et al. [122] propose that ensembles of amyloplasts are mechanically connected by cytoskeletal filaments (or other, as yet undescribed, structures) operating together across several adjacent cells. Gallep et al. [122] also speculate that sensitivity to microscale gravity variation could involve the mesoscopic action of water, since coherently organized and mobile clusters of water could provide electrical-mechanical input to cells in response to conformational or positional changes induced by the gravimetric tide (a notion previously explored by Barlow in 2012 [112]). Water is important not only within cells but also in their cell walls, forming a super-symplasmic continuum that permeates the whole plant body. A further key idea of Gallep et al’s hypothesis is the important role of the orientation of cell walls as the guideline for the structured network. Combining as it does, roots, cell walls, gravity, and cytoskeleton, this represents an elegant integration of several of Peter’s areas of research interest in one testable hypothesis.

These concepts, ideas and findings not only help to push the boundaries of knowledge further and challenge accepted, mainstream wisdom, but also are the very lifeblood of science that enhances our understanding of biological phenomena and the natural world. Furthermore, and although these phenomena may not be viewed as examples of plant neurobiology (see later **Pushing the Boundaries: Plant ‘Neurobiology‘** section), they are a powerful reminder of the range of sensitivities that otherwise sessile, rooted-to-one-spot plants have developed over the course of their evolution that enables them to detect – and respond appropriately to – information in their environment.

## Challenging the *status Quo*

Although outwardly Peter was the very epitome of a quietly-spoken, mild-mannered, genteel Englishman, as a scientist he had what may be considered a maverick streak. This is visibly demonstrated in his work on developing the concept of the Cell Body [124–127]. Originally proposed by Daniel Mazia [15–17], this concept posits that the nucleus, with associated microtubules, represents the basic autonomous unit of eukaryotic life. This cell body pervades the whole eukaryotic cell and becomes the mitotic apparatus during cell division. As a notion it represents a major overhaul of the long-standing Cell Theory [128,129]. Attributed to the work of Schleiden and Schwann [130] – as modifed by Virchow’s notion that cells only arise from pre-existing cells [131] – the Cell Theory has been a cornerstone of biology since it was originally expressed in the late 1830s. Although, as Baluška et al. remind us [126,127], it wasn’t finally and fully accepted until the cellular nature of nerve tissue had been conclusively established in the second half of the 20th Century [132].

The Cell Theory has not been without its problems. Indeed, soon after it was put forward, discoveries such as multinuclear cells, and prokaryotic cells which lacked membrane-bound organelles, challenged its universality and validity [127,133]. It’s probably no surprise therefore that a profound thinker such as Peter turned his attention to this long-established – albeit continually troubled and challenged – idea. Those musings, which were inspired by his cytoskeletal investigations, led to further development of the notion of the Cell Body near the end of the 20th Century [134], although one can already see it being developed in Baluška and Barlow in 1993 [135] and Baluška et al. in 1997 [136].

Notwithstanding, further refinements to the Cell Body concept in further publications, [137,138] an update is considered timely. Accordingly, in the *SIAB*, the Cell Body notion, particularly as it applies to plants, is brought up-to-date – and to the attention of a wider audience – via the Viewpoint from Baluška and Lyons [139]. Considering new discoveries in the biology of eukaryotic cells related to such features as nuclear pores, cilia and flagella, as well as the characterization of archaea from the TACK superphylum [140] with several critical eukaryotic signature proteins, Baluška and Lyons [139] propose a symbiotic origin of the Cell Body in which both the host and guest cells emerge to be of an archaean nature.

## Pushing the boundaries: plant ‘neurobiology‘

The history of science is littered with episodes that may be seen with the benefit and luxury of hindsight as ‘pivotal‘ moments. Unfortunately, one doesn’t necessarily know that they were worthy of that accolade until a long time afterwards. May we be bold and suggest that one such turning point took place just after the start of the current millennium when an article entitled *Plant Intelligence* was published [141]. The author acknowledged that its title and topic was controversial, and the article predictably received a response. That from Firn [142], which presented an alternative point of view, was published in the same journal as the original article of Trewavas, as was the reply thereto by Trewavas [143]. And Peter was amongst the first in the 21st century who sought to promote this more enlightened view of plant biology, that plants were imbued with more sophisticated problem-solving capabilities than they had previoulsy been credited with. This notion eventually led to the establishment of the discipline of plant neurobiology [144–151] and Peter was invited to make presentations at the 1st International Symposium on Plant Neurobiology in 2005, the 3rd in 2007 and 5th in 2009.

Along the way Peter posited the notion that the actin-based cell-cell adhesion domains in the root apices resembled neuronal synapses [152,153], and revived Charles Darwin’s notions of brain-like properties of roots [154,155]. And Peter didn’t shy away from discussing one of the most contentious aspects of plant intelligence, whether plants exhibit consciousness [156,157]. For the record, Peter was of the view that both non-human animals as well as plants exhibit a *protoconsciousness* [158], whereas Man has developed further capabilities, and is able to experience a ‘higher’ state of consciousness [158]. But Peter was also keen to try and broaden awareness of, and encourage discussion about, matters such as cognition and intelligence in the context of plants, especially roots. That aspiration is cited as one of the reasons behind his paper that regarded roots as autopoietic and cognitive constructions [150].

Whilst it must be acknowledged that notions of plant intelligence remain somewhat controversial and contested [159–164], much of this debate seems more to do with concerns over the terminology and language used to describe these plant phenomena rather than denial that plants exhibit complex problem-solving behaviours. Certainly, the finding that plants may exploit ‘alternative means of communication‘ and plant learning [165,166], their apparent ability to ‘hear‘ running water [167], and the evidence accumulated in books such as ‘*What a Plant Knows*‘ [162], ‘*Plant Behaviour & Intelligence‘* [163], ‘*Plant Sensing and Communication‘* [168], ‘*Brilliant Green: The Surprising History of Plant Intelligence‘* [169], *‘Memory and Learning in Plants’* [170], and ‘*The Revolutionary Genius of Plants: A New Understanding of Plant Intelligence and Behavior‘* [171], all add to a growing body of work attesting to the extraordinary sensory capabilites of plants.

Peter’s interests in this area of enquiry is represented in the *SIAB* by Yokawa et al. paper [172] which reports on numerous aspects of plant biology that are affected by anaesthetic agents previously considered only to be active in animals. That study reveals that the actions of anaesthetics are similar in plants and animals at the cellular and organ levels. It also suggests that plants are emerging as attractive model objects to study elusive questions related to actions of anaesthetics, and to serve as an alternative test system for human anaesthesia [172,173]. This work adds to the expanding catalogue of studies that testify to fundamental similarities in plant and animal biologies at the chemical levels. For example, GABA (Gamma-aminobutyric acid), a compound that is important as a signalling molecule in the human brain, is increasingly being shown to alter signalling processes in plants [174–176]. As the debate continues as to how intelligent plants are, the data continue to accumulate and demonstrate their remarkable problem-solving properties and behaviours; the discussion is nowhere near complete!

## It’s not just analysis, but synthesis too…

Although Peter was adept at detailed studies that attempted to unravel the minutiae of botanical phenomena, he wasn’t confined to those tiny bits-and-pieces of biology. His vision was bigger than that, and *synthesis* was as important as small-scale *analysis*. Peter had a great interest in the more philosophical side of botany in his reflections on the hierarchical organization of plants [177], and the transfer of information within the organism [178], and between the organism and the environment [179]. We have seen aspects of that in Peter’s wider-reaching contributions to plant ‘intelligence‘ and plant-environment interactions, extending as far as the moon and its influence on the biology of Earth-bound plants. It therefore seems appropriate that the *SIAB* has a contribution by Tran et al. [180] investigating the mode of action of methanol and information transfer'.

Methanol is produced by enzymic breakdown of pectins in plant cell walls in response to such ‘challenges‘ as herbivory and infection by fungi or bacteria [181]. This volatile compound can be detected by neighbouring plants who exploit this information molecule in priming their own defences should they in turn be attacked by the same threat. As important at this process is, the molecular mechanism(s) underlying this methanolic defence-priming are not well understood. Accordingly, Tran et al. [180] investigated the effects of methanol on Arabidopsis and tobacco BY2 cells. They found that methanol induces variations in cytoplasmic concentrations of calcium that could then interact with reactive oxygen species and a signalling pathway that results in already well-characterised plant response to pathogen attack such as plasma membrane depolarization and synthesis of ethylene. Intriguingly, this potential for methanol-induced release of ethylene generates another volatile compound that can be detected by neighbouring plants and affect their biology in turn. Whilst this isn’t plant neurobiology, the notion of cell wall-facilitated ‘communication‘ between plants is an interesting example of how seemingly disparate aspects of plant biology – in this case cell wall chemistry and plant-plant communication – come together and contribute to a more holistic appreciation of plant behaviour. And, as we’ve already seen with the work of Potocka and Szymanowska-Pułka [93] and Dreyer and Edelmann [94] in the *SIAB*, ethylene is fast becoming one of the most important and topical of the classical plant hormones [95].

## Concluding remarks

What this appreciation has no space to cover in any depth are other aspects of Peter’s scientific career. Amongst which are three papers published in probably the world’s most prestigious science journal *Nature*, [126,182,183] his role as an educator with his *Encyclopedia of Life Sciences* article on the primary root [12]; his inventiveness in developing new techniques for study of mammalian chromosomes [182], analysis of the cell cycle in the root quiescent centre [61], and image analysis of root caps [184]; his contributions to studies of the meaning of sleep and dreaming [185]; the relevance of a new hypothesis of pathogenesis to Alzheimer’s disease [186], and possible genetic and epigenetic links between human inner speech, schizophrenia and altruism [187]; and his intriguing paper considering why there are so many sperm cells [188].

In view of his many accomplishments – not just in the botanical field as highlighted in this article, but also in areas such as human health as touched upon above, and other areas as outlined by Chaffey [189] – Peter has been described as a modern-day Renaissance man [190]. This description seems particularly apt in view of Peter’s strong links to the Mancuso laboratory (The International Laboratory for Plant Neurobiology; LINV, 2016) in Florence, which city was arguably the geographic focus of the Renaissance of the immediate post-mediaeval period [191,192]. Indeed, one way to think of Peter is by analogy to the shape of a plant cell, whose biology he studied for so long and in such depth throughout his career. The shape of a plant cell is likened to a tetrakaidecahedron [193,194], a 14-sided geometrical solid. If one were to enumerate all of the different aspects of Peter’s life – and we’ve had no space here to mention his accomplished piano playing, his enviable collection, and knowledge, of the literary works of John Cowper Powys, his melodious bass voice, his translations of work of the Chilean poet Gonzalo Rojas, surrealist Chilean painter Matta, and Mexican poet Octavio Paz, his art work, and cooking prowess – we have far more than 14 sides to Peter’s life. It seems rather fitting that Peter was at least as multi-faceted as his favourite object of study.

**Figure 2.**
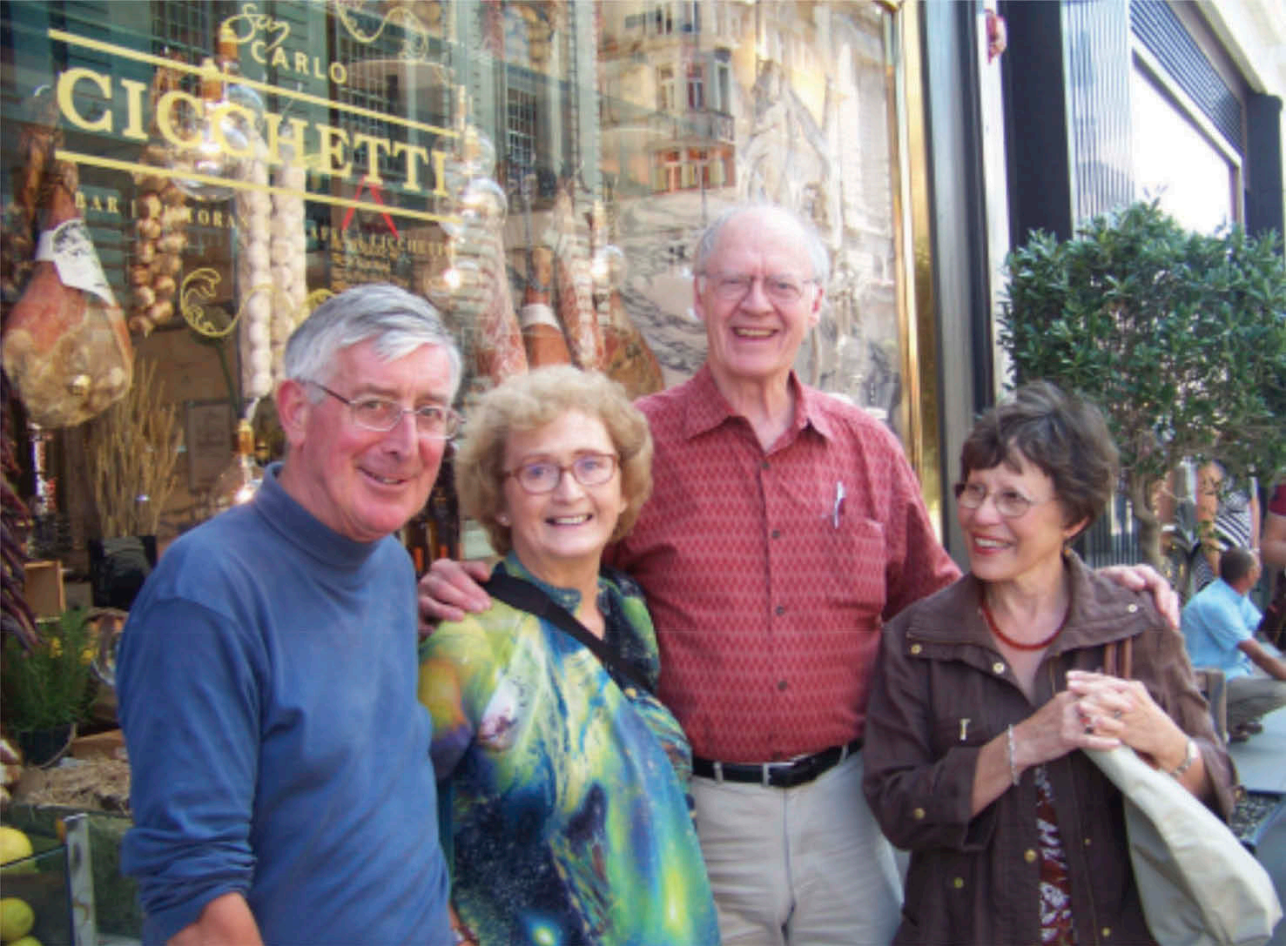
Left to right: Peter Barlow, Marilyn Seago, Jim Seago, and Sonia Barlow outside Cicchetti’s [London] before a visit to the Linnean Society of London.

We conclude this article with the words of Prof. Jim Seago (Figure 2), friend of Peter:

“Peter was probably the botanical world’s most important conceptual thinkers in plant development and structure in the past 50 years, and he backed that up with his researches. Further, his writings represent some of the best, as well as most important, communications in botany and biology, in general. Peter was truly unique!“
